# Potential Effect of Physical Activity Calorie Equivalent (PACE) Labeling on Adult Fast Food Ordering and Exercise

**DOI:** 10.1371/journal.pone.0134289

**Published:** 2015-07-29

**Authors:** Ray Antonelli, Anthony J. Viera

**Affiliations:** 1 School of Medicine, University of North Carolina, Chapel Hill, NC, United States of America; 2 Department of Family Medicine, University of North Carolina, Chapel Hill, NC, United States of America; Cinvestav-Merida, MEXICO

## Abstract

**Introduction:**

Numeric calorie content labels show limited efficacy in reducing the number of calories ordered from fast food meals. Physical activity calorie equivalent (PACE) labels are an alternative that may reduce the number of calories ordered in fast food meals while encouraging patrons to exercise.

**Methods:**

A total of 1000 adults from 47 US states were randomly assigned via internet survey to one of four generic fast food menus: no label, calories only, calories + minutes, or calories + miles necessary to walk to burn off the calories. After completing hypothetical orders participants were asked to rate the likelihood of calorie-only and PACE labels to influence (1) food choice and (2) physical activity.

**Results:**

Respondents (n = 823) ordered a median of 1580 calories from the no-label menu, 1200 from the calories-only menu, 1140 from the calories + minutes menu, and 1210 from the calories + miles menu (p = 0.0001). 40% of respondents reported that PACE labels were “very likely” to influence food item choice vs. 28% for calorie-only labels (p<0.0001). 64% of participants reported that PACE labels were “somewhat likely” or “very likely” to influence their level of physical activity vs. 49% for calorie-only labels (p<0.0001).

**Conclusions:**

PACE labels may be helpful in reducing the number of calories ordered in fast food meals and may have the added benefit of encouraging exercise.

## Introduction

Obesity affects more than one third of adults in the United States and is correlated with health outcomes including hypertension, hyperlipidemia, type 2 diabetes, stroke, and some cancers [1–3]. Moreover, obesity is hypothesized to result in added health care costs that exceed 100 billion dollars annually [4].

In part to combat the rise in prevalence of obesity, the Patient Protection and Affordable Care Act (PPACA) will mandate caloric content posting in restaurants with 20 or more locations [5]. However, evidence suggests that calorie labeling may not have a substantial effect in reducing caloric consumption. One systematic review on the efficacy of caloric content labeling identified 13 relevant studies, two of which were deemed high quality and five of fair quality, with seven total studies included [6]. Only two of the seven studies reported statistically significant reductions in calories purchased, suggesting that calorie-labeled menus have limited influence on the purchasing behaviors of fast food patrons. Subsequent studies have added support to the hypothesis that caloric content labeling has limited efficacy as a strategy to reduce the caloric content of meals ordered [7–9].

Contextual or interpretive representations of caloric content might be more readily understandable to the lay public than menus that show only the number of calories in a given food item. Studied interventions include a “traffic light” system to express the healthiness of a food item and a physical activity calorie equivalent (PACE) label that shows the miles or minutes of walking necessary to burn off the calories in a given food item [10,11]. These measures are hypothesized to reduce the number of calories ordered in a meal by simplifying food choices for consumers who are in a hurry or who might possess limited understanding of caloric labeling. One systematic review and meta-analysis of 17 studies found that alternative representations of caloric content reduced calorie consumption by 81 kcal per meal on average compared to a reduction of 13 kcal for menus labeled only with numeric calorie content [9].

Obesity is associated with a milieu of environmental factors including changing food consumption behaviors and insufficient levels of physical activity [12]. PACE labels address both of these factors and show potential utility in reducing the number of calories consumed by adults during one meal based on results from focus groups and an internet survey [11,13]. Eighty two percent of participants in one survey preferred PACE labeled menus to menus labeled with calories alone [13], and PACE labeling may encourage physical activity by reinforcing the notion that exercise burns calories [11].

**Fig 1:**
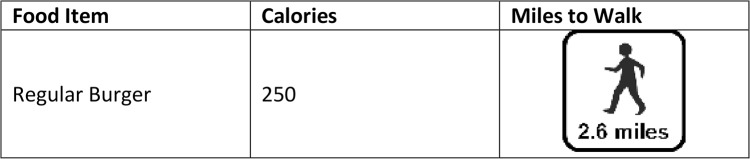
Sample PACE label for a regular burger conveys A.) Caloric content and B.) Miles needed to walk to burn off the calories in the regular burger.

Dowray et al.[13] studied the efficacy of PACE labels via internet-based survey in which 802 participants were randomly selected to order from hypothetical fast food menus showing either (1) food label only, (2) food label and calorie content, (3) food label, calorie content, and minutes to walk to burn off the calories in a given menu item, or (4) food label, calorie content, and miles to walk to burn off the calories in a given menu item (Fig 1). The average number of calories ordered were 1020, 927 (p = 0.14), 916 (p = 0.09), and 826 (p = 0.0007) respectively (p values reported for pairwise comparison to food label-only menu), suggesting that PACE labels may be effective in reducing the number of calories ordered by fast food patrons. That study was limited, however, to a well-educated, predominantly female sample from a small geographic area.

We examined the potential effects of PACE labeling in a national sample. We hypothesized that our results would be similar to those of Dowray et al. [13], with participants shown PACE-labeled menus ordering meals with lower caloric content on average than participants ordering from menus with no labels or with caloric content labels only. We also hypothesized that participants would rate PACE labels as more likely than calorie-only labels to influence both choice of food item and likelihood to exercise.

## Methods

### Label and Menu Design

Labels (Fig 1) were designed and utilized in a prior study [13] and were based on focus group feedback in which participants indicated a preference for caloric content expressed as “miles to walk” or “minutes to walk.” [11] Values were calculated from an activity table for an average 160 pound adult [14] and an assumed walking pace of 30 minutes/mile, which yielded an average calorie burning rate of 3.2 kcal/minute. By dividing the number of kcals in a given menu item by this 3.2 kcal/minute calorie burning rate, the number of minutes needed to walk to burn off a given menu item were calculated. Using this “minutes to walk” value the number of miles needed to walk to expend the calories in a given menu item were also calculated.

The menu used in our survey was identical to the menu used in a previous study [13]. Briefly, the menu items shown to participants were chosen to represent generic items that are available at fast food restaurants nationwide. We included no brand names except the labels for Coke, Diet Coke, and Sprite. A registered dietician reviewed the menu for accuracy and similarity to real-world fast food menus.

### Study Population

This study was granted an exemption from IRB approval by the IRB at University of North Carolina, Chapel Hill (IRB#: 14–0900). Participant consent was not required because data were collected, analyzed, and reported anonymously, and was determined to be exempt from further review according to the regulatory category cited under 45 CFR 46.101(b).

Participants were recruited in August 2014 by Survey Sampling International (SSI). SSI recruited participants from 47 US states via email invitation to pre-existing participant panels and by banner advertisements posted to online communities, social networks, and websites. Potential participants were digitally fingerprinted and checked against third party databases to ensure each respondent was unique. For completing the survey, SSI offered respondents a cash reward ($0.50–$1.00) or entry into sweepstakes for prize drawings. To be eligible for our study participants had to meet the following criteria: 18 years of age or older, parent of at least one child 2 to 17 years old (because we also had a separate aim to examine potential effects on parents’ ordering for children), and had to have eaten at a fast food restaurant in the last month.

Participants were randomized to one of the four study menus by SSI’s software algorithm, which randomly selected and displayed one of the four menus to participants who initiated the survey. After response collection and exclusion we analyzed data from 823 respondents randomized to one of four menus: no labels (n = 189), calories only (n = 209), calories plus minutes to walk (n = 213), or calories plus miles to walk (n = 212). The outcomes we measured included the total number of calories ordered and the total number of calories ordered within specific menu sub-categories (eg. calories from sandwiches, sides, or beverages). We also recorded a number of demographic variables including participant “health literacy,” which we measured with a validated toolkit called Newest Vital Sign [15]. In our survey, health literacy was considered “adequate” if participants answered at least four of six questions correctly and “low/marginal” if they answered three or fewer correctly.

We also included a series of questions to gauge participant attitudes about PACE labels. After completing “orders,” all participants regardless of assigned menu label type (and without ability to go back to change their orders) were shown the three label types and asked to report how likely each label would be to (1) influence their food choices and (2) encourage them to exercise. Responses were constrained to a five point Likert scale ranging from “very likely” to “very unlikely.”

### Data Analysis

SSI did not record responses if participants completed the survey in in less than one-third of the median response time of 16.6 minutes (response not recorded if completed in less than 5.53 minutes). We excluded respondents who ordered meals of zero calories for themselves (n = 4) or their children (n = 7). Responses totaling >4000 calories for adults (n = 155) or their children (n = 11) were also excluded. Several respondents entered values of height and weight corresponding to BMIs incompatible with life (eg; BMI of <5 kg/m2). Therefore, we excluded participants with BMIs more than two standard deviations below the mean BMI (n = 17). We used Kruskal-Wallis to compare the median total number of calories ordered and the number of calories ordered by food category (eg. calories from sandwiches or calories from beverages) across all four menu types. Wilcoxon rank-sum was used to test pairwise differences. We used Stata software (Stata Corp, College Station, TX) for analyses.

## Results

### Participant Characteristics

Comparing demographic characteristics across menu types, no notable differences were found (Table 1). Mean respondent age was 38 years, and the majority of participants reported weights and heights that classified them as being overweight or obese (60%). Most respondents were white (72%) and female (72%). 79% of respondents reported at least some college education while only 55% were found to have “adequate” health literacy. A majority of respondents agreed that they think fast food is a splurge (66%), that they consider the “healthiness” of fast food menu items (57%), and that they are currently trying to lose weight (57%), and most reported having eaten at a fast food restaurant within the past week (77%). Respondents were from all four geographic regions of the United States as defined by the United States Census Bureau[16], with 25% from the West, 22% from the Midwest, 36% from the South, and 17% from the Northeast. A plurality (46%) of respondents described their geographic location as suburban.

**Table 1:** Characteristics of 823 Participants by Menu Type.

	No Label (n = 189)	Calorie Label (n = 209)	Calories + Minutes (n = 213)	Calories + Miles (n = 212)
Age^a^	38 (9.7)	38 (10)	39 (10)	39 (9.6)
Last Fast Food Meal (%)				
Less than 1 week ago	79	77	80	74
Between 1–2 weeks ago	17	19	14	19
About a month ago	4	4	6	7
BMI (kg/m^2^)^a^	26 (7.1)	28 (7.9)	28 (7.9)	28 (8.4)
Sex (%)				
Male	23	28	34	24
Female	77	72	66	76
Education^b^ (%)				
High School	19	25	22	17
At least some college	64	61	61	70
Graduate Degree	17	14	17	13
Total Annual Household Income^c^ (%)				
Less than $35,000	23	25	24	29
$35–75,000	41	39	45	36
More than $75,000	33	32	29	32
Geographic Setting (%)				
Rural	15	19	17	19
Small Town	9	11	14	14
Suburban	44	49	46	46
Urban	29	20	22	21
Unsure	3	1	2	0
Fast Food a Splurge? (%)				
Agree	63	65	64	72
Disagree	25	25	26	18
Unsure	12	10	10	10
Consider “Healthiness” of Fast Food Items (%)				
Agree	60	59	50	58
Disagree	33	28	39	33
Unsure	7	13	11	9
Currently Trying to Lose Weight? (%)				
Agree	53	55	57	63
Disagree	42	39	37	32
Unsure	5	6	6	5
Race/Ethnicity^d^ (%)				
Black	13	13	8	7
White	64	73	73	75
Hispanic	10	7	10	7
Other	13	7	8	11
Geographic Region (%)				
West	24	23	26	25
Midwest	15	22	24	25
South	40	39	34	32
Northeast	21	16	16	18
Health Literacy^e^ (%)				
Adequate	49	56	59	56
Inadequate	51	44	41	44

^a^Mean (SD)

^b^One participant selected “prefer not to answer”

^c^22 participants selected “prefer not to answer” or “do not know”

^d^6 participants selected “prefer not to answer”

^e^Adequate health literacy defined as a score of ≥4 on Pfizer’s Newest Vital Sign

### Calories Ordered

There was a significant difference in the median total calories ordered across menu types, a trend that was also observed in most menu subtypes (Table 2). Participants shown menus without calorie labels ordered a median of 1580 calories compared to participants shown menus with calories only (1200), calories plus minutes to walk (1140), or calories plus miles to walk (1210; p = 0.0001). Pairwise comparisons between calories only, calories+minutes, and calories+miles label types were non-significant.

**Table 2:** Calories (Median) Ordered by Menu Type.

	No Label	Calories Only	Calories + Minutes	Calories + Miles	P value
Total Calories Ordered	1580	1200*	1140*	1210*	0.0001
Calories from Burgers	390	360	380	360	0.006
Calories from Sides	380	380	380	230	0.007
Calories from Salads	190	110	110	190	0.027
Calories from Drinks	210	150	190	150	0.0008
Calories from Desserts	160	0*	150	70	0.0001

*Pairwise comparisons to no label: P<0.0001

Statistically significant differences were observed across menu label types in most demographic subcategories (Table 3). Obese participants ordered fewer calories from labeled menus. No significant associations were observed in participants reporting household income of less than $35,000, in Hispanics, and in participants from Western and Mid-Western geographic regions.

**Table 3:** Calories (Median) Ordered by Demographic Subgroup.

	No label	Calories Only	Calories + Minutes	Calories + Miles	P value
Sex					
Males	1945	1250	1230	1320	0.004
Females	1540	1185	1120	1180	0.0003
BMI (kg/m^2^)					
<25	1605	1240	1220	1325	0.033
25–29.9	1480	1110	1100	1140	0.025
≥30	1840	1140	1140	1130	0.027
Education Level					
High School	1770	1165	1320	1280	0.093
At least some college	1520	1220	1110	1130	0.001
Graduate Degree	1760	1160	1225	1465	0.006
Household Income					
Less than $35,000	1525	1230	1180	1400	0.52
$35–75,000	1745	1180	1225	1140	0.0001
More than $75,000	1380	1205	1020	1160	0.036
Race/Ethnicity					
Black	2055	1140	1260	1400	0.001
White	1480	1135	1090	1130	0.001
Hispanic	1510	1670	1300	1345	0.66
Other	1750	1315	1680	1275	0.14
Health Literacy					
Adequate (score ≥4)	1450	1165	1070	1090	0.005
Inadequate (score <4)	1750	1220	1350	1420	0.002
Geographic Region					
West	1580	1190	1080	1300	0.15
Midwest	1670	1130	1140	1180	0.17
South	1510	1230	1120	1090	0.006
Northeast	1670	1130	1300	1330	0.045

P-values by Kruskal Wallis

### Label Influence on Self-Reported Likelihood of Behavior Change

**Fig 2:**
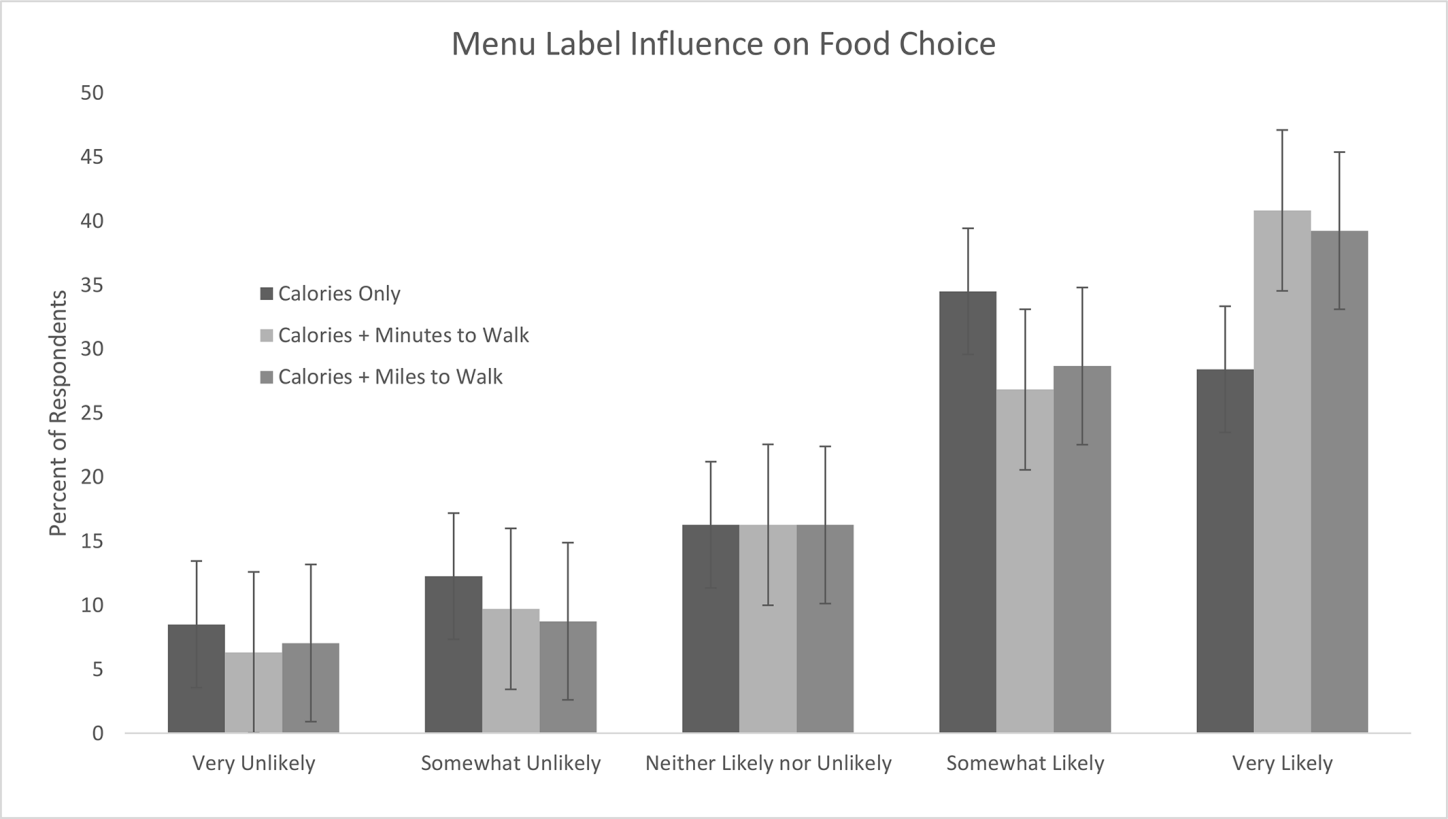
Self-reported likelihood of PACE labels to influence participant food choice.

**Fig 3:**
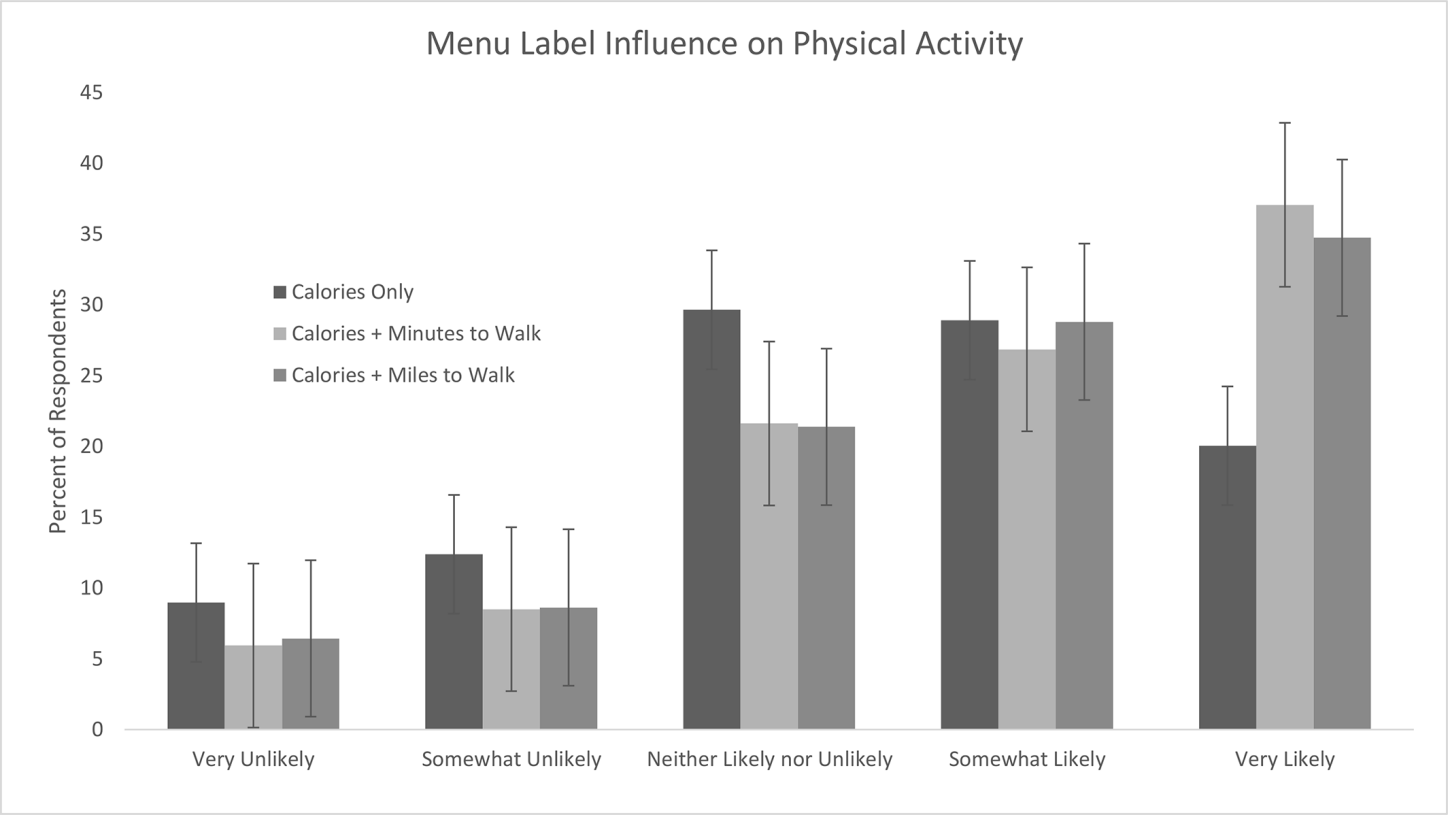
Self-reported likelihood of PACE labels to influence participant physical activity level.

Participants reported that PACE labeling would influence both their food choices (Fig 2) and their likelihood of engaging in physical activity (Fig 3). 28% of respondents reported that a label with calories only was “very likely” to influence their choice of food item, compared to 41% (minutes to walk) and 39% (miles to walk) of respondents who reported that PACE labels would be “very likely” to influence their food choice (p<0.0001). Similarly, 64% of respondents rated both minutes to walk and miles to walk labels as “somewhat likely” or “very likely” to encourage them to engage in physical activity compared to 49% of people shown the calories-only label (p<0.0001).

## Discussion

### PACE Labels and Calorie Reduction

Our findings suggest that in a national cross-sectional sample of individuals ordering from hypothetical fast food menus, caloric content labels influence participants to place lower-calorie orders. This effect was observed equally in groups shown menus labeled only with calories and in groups shown PACE labels. In contrast, other studies have shown alternative labeling schemes to be more effective than numeric calorie labels alone. These alternative labels have included PACE labeling [13], energy equivalent labeling [17], traffic light labeling [8], and menus that include total daily caloric content recommendations [18]. Additionally, PACE labels may show benefit in influencing the behavior of parents ordering fast food for their children [19].

A desirable characteristic of any menu labeling intervention is to demonstrate efficacy in a wide range of demographic groups. Our results suggest that PACE labeling is effective in most subgroups. Those subgroups in which labeling schemes showed no statistically significant benefit (Hispanics, individuals reporting household income <$35,000, and residents of the West and Midwest) were likely constrained by sample size. Importantly, calorie-only and PACE menu labeling were associated with a statistically significant decrease in the number of calories ordered across categories of sex, education level, and health literacy.

### PACE Labels and Likelihood of Behavior Modification

Previous research suggests that even small changes in food labeling may have significant public health benefits [20]. Although we found calorie-only labels to be equally effective as PACE labels in reducing the total number of calories ordered, more respondents reported that PACE labels were “very likely” to influence their food choices. More respondents also reported that PACE labels were “very likely” or “somewhat likely” to encourage them to engage in physical activity. We were unable to ascertain whether PACE labels influenced participants’ actual physical activity levels. In a real-world setting, even in the absence of calorie reduction, a labeling scheme that encourages exercise could prove beneficial. Further research is needed to assess the efficacy of PACE menu labels in a real world setting to both reduce the number of calories ordered and to encourage restaurant patrons to exercise.

### Study Limitations

The major limitation of our study is that our survey was internet-based and participants completed orders that were hypothetical. Thus, we could not account for variables such as hunger, time pressure, costs, and marketing, which would likely influence real-world behavior. The external validity of our sample may be further limited by the high proportion of female respondents, the hypothetical nature of the study, and the potential selectivity bias introduced by the necessity of an internet connection to complete the survey.

Another limitation of our study was the number of participants who ordered meals in excess of 4000 calories (n = 155) including some who ordered more than 15,000 calories. Meals of this size are rarely ordered in reality, and similarly, even among participants included in our survey average calorie counts seemed higher than real-world settings [21]. Possible explanations for this phenomenon include participant keying error, arbitrary menu item selection, or participants who mistakenly believed they were required to choose items from all menu subsections. Additionally, participants may have ordered more food than usual because prices were not included in the survey.

A real-world study could address these limitations. Participants would expect to eat what they order, eliminating the problem of unrealistically high-calorie meals. A real world setting would introduce additional variables that do not exist during an internet-based survey. These variables may include time constraint, hunger, and marketing, and PACE labels may simplify decision-making in these situations. Furthermore, our results suggest that PACE labels are more likely than calorie-only labels to influence both food choices and physical activity levels. A real world study could have the added benefit of measuring participant physical activity levels following introduction of PACE labels.

## Conclusions

Both calorie-only and PACE menu labels appear effective in reducing the number of calories participants order from hypothetical fast food menus. PACE labels may have the added benefit of encouraging restaurant patrons to exercise. Further research is warranted to study the efficacy of PACE menu labels in real-world settings.
